# Elucidating the Multi-Targeted Role of Nutraceuticals: A Complementary Therapy to Starve Neurodegenerative Diseases

**DOI:** 10.3390/ijms22084045

**Published:** 2021-04-14

**Authors:** Tapan Behl, Gagandeep Kaur, Aayush Sehgal, Sukhbir Singh, Saurabh Bhatia, Ahmed Al-Harrasi, Gokhan Zengin, Simona Gabriela Bungau, Mihai Alexandru Munteanu, Mihaela Cristina Brisc, Felicia Liana Andronie-Cioara, Ciprian Brisc

**Affiliations:** 1 Department of Pharmacology, Chitkara College of Pharmacy, Chitkara University, Chandigarh 160009, India; tapanbehl31@gmail.com (T.B.); kgagandeep060@gmail.com (G.K.); aayushsehgal00@gmail.com (A.S.); sukhbir.singh@chitkara.edu.in (S.S.); 2 Natural and Medical Sciences Research Center, University of Nizwa, 616 Birkat Al Mauz, P.O. Box 33, Nizwa, Oman; sbsaurabhbhatia@gmail.com (S.B.); aharrasi@unizwa.edu.om (A.A.-H.); 3 Department of Biology, Faculty of Science, Selcuk University Campus, Konya 42130, Turkey; biyologzengin@gmail.com; 4 Department of Pharmacy, Faculty of Medicine and Pharmacy, University of Oradea, 410028 Oradea, Romania; 5 Department of Medical Disciplines, Faculty of Medicine and Pharmacy, University of Oradea, 410073 Oradea, Romania; mihaimunteanual@yahoo.com (M.A.M.); briscristina@yahoo.com (M.C.B.); brisciprian@gmail.com (C.B.); 6 Department of Psycho-Neuroscience and Recovery, Faculty of Medicine and Pharmacy, University of Oradea, 410073 Oradea, Romania; felicia_cioara@yahoo.com

**Keywords:** nutraceuticals, neurodegenerative diseases, oxidative stress, mitochondrial dysfunction, apoptosis, neuroprotective, complementary therapy

## Abstract

The mechanisms underlying multifactorial diseases are always complex and challenging. Neurodegenerative disorders (NDs) are common around the globe, posing a critical healthcare issue and financial burden to the country. However, integrative evidence implies some common shared mechanisms and pathways in NDs, which include mitochondrial dysfunction, neuroinflammation, oxidative stress, intracellular calcium overload, protein aggregates, oxidative stress (OS), and neuronal destruction in specific regions of the brain, owing to multifaceted pathologies. The co-existence of these multiple pathways often limits the advantages of available therapies. The nutraceutical-based approach has opened the doors to target these common multifaceted pathways in a slow and more physiological manner to starve the NDs. Peer-reviewed articles were searched via MEDLINE and PubMed published to date for in-depth research and database collection. Considered to be complementary therapy with current clinical management and common drug therapy, the intake of nutraceuticals is considered safe to target multiple mechanisms of action in NDs. The current review summarizes the popular nutraceuticals showing different effects (anti-inflammatory, antioxidant, neuro-protectant, mitochondrial homeostasis, neurogenesis promotion, and autophagy regulation) on vital molecular mechanisms involved in NDs, which can be considered as complementary therapy to first-line treatment. Moreover, owing to its natural source, lower toxicity, therapeutic interventions, biocompatibility, potential nutritional effects, and presence of various anti-oxidative and neuroprotective constituents, the nutraceuticals serve as an attractive option to tackle NDs.

## 1. Introduction

Neurodegenerative disorders/diseases (NDs) are a chronic debilitating group of heterogeneous diseases, which include loss of neuronal function and structure, leading to neuronal cell death or progressive degeneration [1,2,3]. NDs comprise a highly complex etiology that is mainly associated with abnormal protein accumulation, mutated genes, increased reactive oxygen species (ROS), neuroinflammation, mitochondrial dysfunction, apoptosis, elevated endoplasmic reticulum (ER), calcium overload, excitotoxicity, or neuronal destruction in specific regions of the brain [1,4]. ND is a wide array of neurological disorders that generally affect central nervous system (CNS) neurons, characterized by progressive neuronal dysfunction in the CNS, resulting in deficit of specific functions of the brain (movement, memory, and cognition). These processes are involved in the pathogenesis and progression of NDs, such as Huntington’s disease, Alzheimer’s disease, amyotrophic lateral sclerosis, multiple sclerosis, and Parkinson’s disease [5,6].

The incidence and prevalence have risen dramatically with >10 million people suffering from NDs annually, and they are expected to rise for the foreseeable future, having direct influence on the socioeconomic status of any country [4,7,8]. Moreover, NDs are extremely associated with ageing, wherein the prevalence rate is higher in older persons than younger persons, owing to multifaceted pathologies [1,2,9]. The prevalence of NDs in western countries is higher in individuals aged 70–79 years than in India (incidence is 0.7%) due to varying lifestyle (sedentary lifestyle and food habits). The therapeutic approach of multifaceted and complex NDs is always a challenge; however, the current treatment options do not counteract the pathogenic processes and are often less effective over a period of time [1,9].

Recently, extensive studies have endeavored to elucidate the growing interest of nutraceuticals as a collective approach to the standard pharmacological therapy, affecting one or more of the aforementioned pathogenic processes or pathways [6,10]. The nutraceutical has no universally accepted definition. However, it represents the association between nutrition and pharmaceutical agents as it encompasses all naturally occurring products (food, functional or fortified food, food components, or nutrients) [11]. The nutraceutical-based unconventional approach is becoming increasingly common due to multiple mechanistic pathways or specific mechanisms of action in NDs. The benefits of nutraceuticals have opened doors to use them as a complementary therapy to tackle and starve NDs [12,13]. Thus, multifactorial pathogenic nature of nutraceuticals targets different mechanisms, i.e., neuroinflammation, ER stress, mitochondrial dysfunction, OS, protein misfolding, and so on. Nutraceuticals in NDs have been able to position themselves as a better and safer integrative approach due to the fact that naturally occurring compounds have fewer side effects and can be combined with common drug therapy to enhance the patient’s quality of life in most cases [4,6].

The current review emphasizes the effectiveness of several nutraceuticals through multiple pathogenic pathways involved in NDs and focuses on the rationale behind their use as a complementary therapy in NDs. Moreover, the manuscript raises the reader’s awareness of present evidence on nutraceuticals that have exhibited the ability to act as neuro-protectants and hold promise as a prophylactic treatment for NDs.

## 2. Brief Overview of Neurodegenerative Diseases (NDs)

ND is a universal terminology for a varied debilitating and often irregular group of disorders categorized by diseases of the dynamic nervous system emerging from neuronal loss and neuronal degradation [14,15]. Apart from neural loss, these NDs are characterized by impaired electro-physiological and neuro-chemical properties in the vulnerable regions of the brain, thus influencing the associated functions [16,17]. Moreover, ND encompasses a wide array of neurological disorders that generally affect CNS neurons, characterized by progressive neuronal dysfunction in the CNS, resulting in deficit of specific functions of the brain (movement, memory, and cognition) [5,6]. NDs comprise a highly complex etiology, mainly associated with abnormal protein accumulation, mutated genes, increased ROS, neuroinflammation, mitochondrial dysfunction, apoptosis, elevated ER stress, calcium overload, excitotoxicity, or neuronal destruction in specific regions of the brain [1,4]. Huntington’s disease, Alzheimer’s disease, amyotrophic lateral sclerosis, multiple sclerosis, and Parkinson’s disease are examples of NDs.

Considered to be second most persisting/recurring ND worldwide with multifaceted etiologies, Parkinson’s disease affects approximately 1–2% of the population [18,19]. An intra-neuronal buildup of protein aggregates and Lewy bodies (consisting of misfolded α-synuclein) and gradual decline of dopaminergic neurons in the substantia nigra pars compacta [20,21] are the two major neuropathological processes involved in Parkinson’s disease. Patients with Parkinson’s suffer from a variety of non-motor symptoms, uncontrollable tremors, and diminished motor function that exacerbate in the later stages of the disease [22,23]. Symptoms include anxiety, dementia, depression, optical delusion, and excessive daytime sleepiness [24]. Several other symptoms may involve cognitive and autonomic dysfunction, olfactory functional loss, and rapid eye movement, which may appear at later stages of this disorder [25].

Alzheimer’s disease is the most common type of ND in the elderly, which worsens with time. Becoming a major healthcare challenge of 21st century, its prevalence continues to rise worldwide. It is the most established cause of dementia characterized by gradual cognitive impairment [26]. Thought to start 20 years or more before, the symptoms arise with brain alterations that remain unnoticeable to the affected person. Individuals after years of brain alterations experience noticeable symptoms (i.e., language problems and memory loss) [26,27,28,29]. The occurrence of symptoms is due to damage or destruction of nerve cells (neurons) in brain regions involved in learning, thinking, and memory [26,30,31]. Therefore, Alzheimer’s disease is characterized by visual-spatial disorders, cognitive functional loss, memory and language impairment, and complications with judgment or analysis. The two proposed fundamental pathogenic mechanisms are the amyloid cascade and the tau hyperphosphorylation [32]. The neuropathological feature includes accumulation of β-amyloid protein, leading to hard plaques interfering with acetylcholine initiating inflammatory processes and affecting synaptic transmission, which may further result in the specific protein (tau) leading to cell demise in Alzheimer’s disease. Therefore, the pairing of microtubules with other tubules of the neurons produces neurofibrillary tangles, resulting in disintegration of tubule and blockade of neurotransmitters, which causes cell death [33,34].

Another progressive ND, amyotrophic lateral sclerosis, is characterized by progressive motor neurodegeneration and death of lower and upper motor neurons in the spinal cord, brain, and brainstem, affecting voluntary muscles and leading to paralysis and respiratory failure and even death [35]. The causative mechanisms underlying amyotrophic lateral sclerosis remain unclear but numerous factors have been included such as excitotoxicity, genetic factors, autoimmune response, protein misfolding and aggregation, oxidative stress, deficits in neurotrophic factors, neurofilament aggregation, environmental factors, mitochondrial dysfunction, and impaired axonal transport [36,37]. This disease is linked with gene mutation that produces superoxide dismutase-1 enzyme. Moreover, this ND is linked with protein inclusions, composed of cytoplasmic trans active response DNA binding protein-43 (TDP-43) in the affected regions of spinal cord and brain [38].

Huntington’s disease is an inherited ND characterized pathologically by diminished functions of gamma-aminobutyric acid and excessive dopaminergic activity in the basal ganglia. Clinically, it is characterized by psychiatric disturbance, abnormal movements, and cognitive deficits [21], being associated with the expansion of the trinucleotide repeat in the Huntington (Htt) gene present at the short arm of chromosome 4. Htt (mutant) proteolysis has been observed to contribute to its pathology, but its role is not yet well defined [39].

An inflammatory, autoimmune, and chronic in nature, multiple sclerosis is a CNS disease characterized by axonal preservation with demyelinated regions. Multiple sclerosis is mostly observed in individuals 20–45 years of age in contrast with other old-age-related NDs [40]. The major driver of pathology in multiple sclerosis is inflammation of CNS. Various factors encourage its development, yet its cause is not well-defined. In MS, the disrupted BBB elicits infiltration of peripheral blood leukocyte, followed by myelin degradation, axonal disruption, and cell loss of neurons. Approximately 2.3 million people are estimated to live with this disease worldwide. Moreover, the prevalence and incidence of this ND is increasing. There are progressive and relapsing–remitting forms of multiple sclerosis; its cause is still not well understood and established [41,42].

## 3. Mechanisms Involved in Neurodegeneration

**Figure 1:**
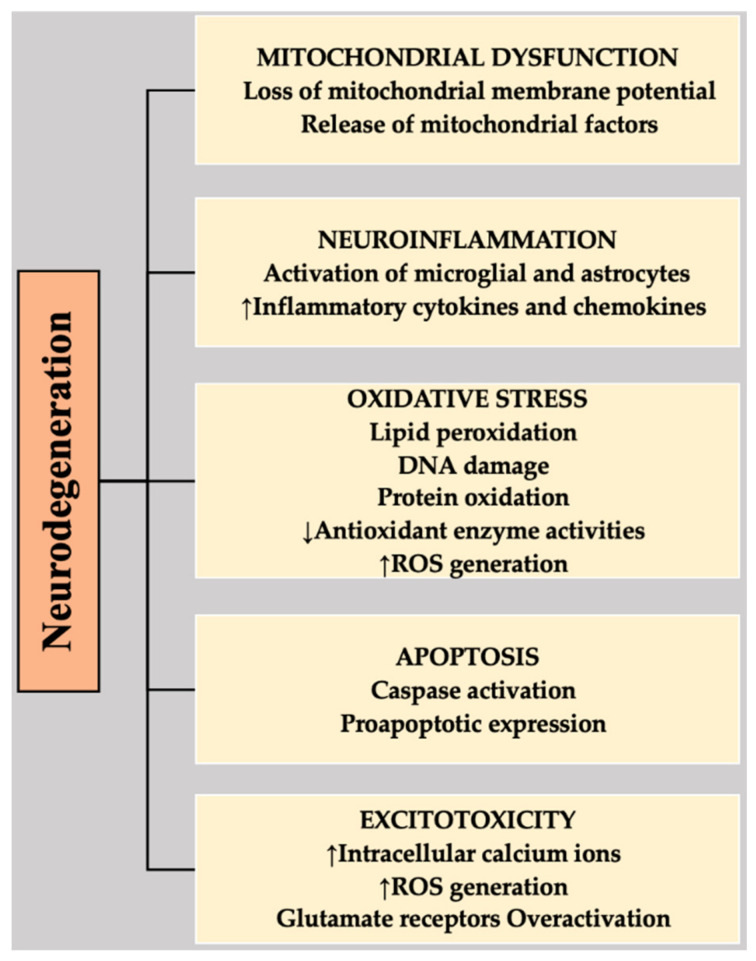
Highlighting the associations of different mechanisms with the neurodegeneration that is involved in NDs. DNA, deoxyribonucleic acid; ROS, reactive oxygen species.

Different mechanisms have been implicated in the progression and pathogenesis of NDs, including OS, neuroinflammation, apoptosis, excitotoxicity, and mitochondrial dysfunction [43,44,45,46,47] (Figure 1).

### 3.1. Apoptosis

Apoptosis facilitates the precisely programmed natural neuronal death that is physiologically essential in neurogenesis during CNS maturation. The process is energy-dependent, which necessitates ATP for signal activation and protein synthesis [48]. Apoptosis is a complicated process, which is triggered by various extrinsic and intrinsic signals. Extrinsic and intrinsic pathways consist of death receptor activation upon ligand binding and production of pro-apoptotic factors in cytosol, respectively. Subsequently, the intrinsic pathway triggers caspase-independent apoptosis or caspase-dependent apoptosis [46,48]. Characterized by chromatin condensation, cell shrinkage, membrane cell death, and DNA fragmentation, neuronal apoptosis is the neuropathological hallmark of NDs. Therefore, abnormality in the regulation of apoptosis or premature apoptosis is involved in the pathogenesis of neurodegeneration, a multifarious process that leads to several NDs, such as Huntington’s disease, Alzheimer’s disease, amyotrophic lateral sclerosis, multiple sclerosis, Parkinson’s disease, etc. [49].

Various primary brain regions undergo neuronal loss; as diseases progress, these regions can be expanded in several conditions. The most noticeable clinical symptom in patients of Alzheimer’s disease is memory loss, which is associated with neuronal loss in the hippocampal region. As Alzheimer’s disease advances, the cortical/subcortical areas experience neuronal losses that can be extremely severe [50,51]. In the apoptosis context, it has been indicated that executor or initiator caspases are activated in Alzheimer’s disease. Moreover, the levels of extrinsic apoptotic pathway protein have been reported to elevate in the brains of Alzheimer’s disease [52]. In the brains of Parkinson’s disease, it has long been proposed that apoptosis is the preferred pathway for the elimination of dopaminergic neurons in substantia nigra. It has been suggested that nearly every Lewy body neuron is positive for pro-apoptotic process, proposing that heavily burdened neuron with protein aggregates undergo apoptosis [53,54]. In Huntington’s disease, it has been specifically proposed that hyperpolarization of mitochondrial membrane occurs via an mHtt monomer to promote apoptotic neuronal death [55]. In amyotrophic lateral sclerosis, it is TDP-43 that was found to induce the pro-apoptotic proteins expression in a p53-dependent manner. However, various studies suggested that loss of neurons in the brains of amyotrophic lateral sclerosis is a caspase-independent process [56,57].

### 3.2. Oxidative Stress (OS)

OS is a contributory hallmark in the pathophysiology of common NDs. OS is the imbalance of ROS production and antioxidative defense ability, resulting in cellular damage and DNA repair system damage, which will speed up the neurodegenerative process and progression of NDs [43].

The nervous system is mainly susceptible to OS for the following reasons:

- lower endogenous antioxidants reserve levels;
- more oxygen consumption due to higher ATP demand;
- availability of polyunsaturated lipids that are mainly vulnerable to attack of free reactive species in the neuronal cell membrane;
- presence of neurotransmitters and excitatory amino acids, the metabolism of which can generate ROS; and
- elevated levels of transition metals [58,59].

These aforementioned characteristics make neuronal cells a target for the damage caused by ROS. In patients with amyotrophic lateral sclerosis, Aβ neurotoxicity and neuronal degeneration are correlated with the oxidative damage to lipids and proteins, DNA, and RNA [60,61]. In Parkinson’s disease, oxidative damage to proteins and DNA has been reported in the nigro-striatal regions [62,63]. Dopamine oxidation to 6-OHDA (reactive) stipulates an essential endogenous cascade for creating Parkinson’s disease-like condition [64]. Moreover, the overload of transition metals (such as iron) has been implicated in the Lewy body formation process in Parkinson’s [65]. OS-induced damage to DNA, proteins, and lipids and mutations in the SOD-1(Cu/Zn)-encoding gene have been linked with sporadic and familial amyotrophic lateral sclerosis forms [66]. The pathology of multiple sclerosis is not dissimilar when the harmful influence of OS is considered. In addition, reactive species generated by the mononuclear cells and activated microglia play a critical role in the pathogenesis of multiple sclerosis. Declined endogenous antioxidants levels and oxidative damage to mtDNA are related to the axonal injury and demyelination in multiple sclerosis [67]. Exacerbation of lipofuscin and increased occurrence of the oxidative DNA strand breaks is known to be associated with Huntington’s disease [68,69]. It has been found that a substantial increase in 8-OH-dG levels occurs in nDNA in the postmortem tissue of Huntington’s disease subjects [70]. All the aforementioned studies noticeably specify a causal and evidential relationship between OS and neuronal cell demise in NDs.

### 3.3. Calcium Overload and Excitotoxicity

Excitotoxicity is another process implicated in the pathogenesis of NDs. It is caused by the persistent over-activation of glutamate receptors by excitotoxins or excitatory amino acids in the CNS, leading to neuronal death [43]. Pathologically elevated levels of glutamate and binding of excitotoxins with the glutamate receptors can cause or trigger excitotoxicity by permitting speedy entry of calcium ions (Ca^2+^) in the cell [71]. In the cell, Ca^2+^ influx activates numerous Ca^2+^-dependent enzymes, including endonucleases, protein phosphatases, lipases, endonucleases, xanthine oxidase, proteases, phospholipases, protein kinase, and inducible nitric oxide synthase (iNOS). These enzymes destroy and damage cellular structures such as membrane, cytoskeleton components, and DNA.

The excessive influx of Ca^2+^ could also render mitochondrial dysfunction, ROS production, OS, and inflammatory responses, ultimately leading to neuronal cell demise [15,72]. Apart from these processes, accumulation of Ca^2+^ in neurons can also take place via different routes such as activation of receptor-operated Ca^2+^ channels, voltage-sensitive Ca^2+^ channels, ATP-dependent Ca^2+^ channel (P2j receptor), Ca^2+^ channels coupled to G-protein receptors, and cyclic nucleotide-gated Ca^2+^ channels [71,73]. An Aβ-induced neuronal cell demise in AD is linked with dysregulation of pathways dependent on Ca^2+^ [74]. In Parkinson’s disease, intracellular Ca^2+^ dysregulation can describe the destruction of dopaminergic neurons [75]. Besides, neuronal injury and destabilization in multiple sclerosis are due to over-activation of calpain and Ca^2+^-dependent protein phosphatases and proteases [76]. The opening of mitochondrial permeability transition pore due to Ca^2+^ release and mitochondrial Ca^2+^ overload from the endoplasmic reticulum by mHtt is considered as the process of neuronal demise in Huntington’s disease [77].

### 3.4. Neuroinflammation

Emerging threads of evidence underscore the role of neuroinflammation in the progression or pathophysiology of NDs. The beginning and consequent increase in neuroinflammation seem to depend on cross-talks among glia, neurons, and immune cells. Macrophages are located in the brain region nearby glial cells that play an essential role in neuroinflammation-mediated NDs. Moreover, activated microglia in the diseased state mediates neuronal injury via release of pro-inflammatory factors. The production of pro-inflammatory factors results in trans-endothelial migration of immune cells across the blood–brain barrier (BBB) [47].

Different processes have been implicated for the actions mediated by microglia that cause neuronal destruction. Phagocytic oxidase (PHOX)-mediated OS-induced neurotoxicity is the foremost mechanism involved here. When PHOX is triggered by inflammatory state, it produces OS by promptly generating high superoxide levels. The inflammatory PHOX activation also stimulates activated microglia leading to production of IL-1β and TNF-α [78,79]. Therefore, activation of PHOX offers an essential link between OS and inflammation [78,80]. Microglia-expressed inducible nitric oxide synthase (iNOS) and astrocytes are upregulated during neuroinflammation, leading to increase production and, thus, promoting neuronal death [43]. However, it should be observed that activation of either NO or NADPH oxidase via iNOS expression alone is insufficient to cause neurotoxicity, but rather their mutual activation triggers the cascade of inflammatory neurodegeneration [78,81]. Moreover, chemokines released by astrocytes exert multifaceted roles in the pathophysiology of chronic NDs including Alzheimer’s disease [43,82].

### 3.5. Mitochondrial Dysfunction

Considered as the site of cellular respiration and oxidative phosphorylation, mitochondria play an essential role in sustaining a low Ca^2+^ concentration in the cytosol. Excessive Ca^2+^ uptake and ROS generation cause the failure of mitochondrial membrane potential and mitochondrial permeability pore opening [83]. The pathophysiology of numerous NDs such as Huntington’s disease, Alzheimer’s disease, amyotrophic lateral sclerosis, multiple sclerosis, and Parkinson’s disease mainly comprises damaged mitochondria [44,83]. Several molecular mechanisms dependent on mitochondria such as generation of ROS, inhibition of mitochondrial electron transport chain (ETC) complexes, dysfunction of the enzymes implicated in the cycle of tricarboxylic acid, perturbations of mitochondrial clearance mechanisms, and impairment of mitochondrial dynamics could promote the pathogenesis of NDs and neuronal injury [44]. In the substantia-nigra, defective ETC (Complex I) is known as the chief cause of sporadic Parkinson’s disease [84]. Mitochondria abnormalities also trigger the neuronal loss and damage in amyotrophic lateral sclerosis and Alzheimer’s disease [85,86]. In multiple sclerosis, neuronal degeneration is also considered to contribute to the irregular activities of ETC (Complexes I and IV) [87]. Several pre-clinical and clinical lines of evidence from Huntington’s disease cases suggest that mitotoxicity plays a critical role in the pathogenesis of neurodegeneration [88]. Owing to complicated cross-talks among mitotoxicity, chronic inflammatory state, and OS, it is not yet well-defined whether damage to mitochondria is the consequence or cause of neuronal dysfunction and damage [89].

## 4. Nutraceuticals and Its Classification

Underlying the category of non-specific biological therapy, nutraceuticals are used in the management and prevention of symptoms of mild disorders to extremely toxic malignancy [4].

First coined by Stephen L DeFelice, “nutraceutical” is the combined term derived from “pharmaceutical” and “nutrition”. As there are different definitions of nutraceuticals, they are mainly considered as any dietary supplements that do not supply calories and elicit health or medical advantages as well as aid in treatment and/or prevention of disease. However, recently, a nutraceutical has been redefined as “food product or its secondary metabolites, which could provide health advantages (to treat and/or prevent particular disease) in the clinical setting” [90]. These have been clinically validated with practical scientific evidence to support their beneficial effect in the prevention or treatment of a particular disease [91,92], being considered as well the main reason that nutraceuticals are accepted by the general public as a treatment form, offering better tendency to believe that there are lesser side effects in comparison to several known synthetic drug compounds [1,2]. The complicated and multifaceted structures as well as variability of nutraceuticals resulted in several categorizations [92]. The recent and most particular classification of nutraceuticals depends on their novelty as follows.

Traditional/food-based nutraceuticals are simply natural foods, which have not been changed, other than the way consumers perceive them. They are simply whole foods associated with potential health benefits. These include grains, vegetables, fruits, meat, fish, dairy, and eggs that offer numerous benefits including basic nutrition. The chemical constituents (nutrients, phytochemicals, herbals/extracts, polyunsaturated fatty acids (PUFAs), probiotics and prebiotics, and nutraceutical enzymes) are the subcategories that fall under this category [93].

Nutrients are defined as feed constituents at a level that supports life. These include many available nutrients, which are primary metabolites exhibiting nutritional properties such as amino acids, minerals, fatty acids, vitamins, and antioxidants. Nevertheless, antioxidants and vitamins are the most commonly used nutrients [94]. Considered to be secondary metabolites, phytochemicals are chemical constituents extracted from plants that offer specific biological effects. They are well-known to exert biochemical reactions and influence the metabolic activity [94,95]. The categorization mainly depends on their chemical structures considering also their actions and roles, including isoflavonoids, polyphenols, anthocyanidins, phytoestrogens, carotenoids, terpenoids, limonoids, glucosinolates, phytosterols, and polysaccharides [95,96]. They affect the body by several mechanisms such as inhibition of enzymatic reactions, acting as cofactor or catalyst and substrate for biochemical reactions, enhance nutrient absorption, toxin elimination, and binding to cell receptors exhibiting antagonistic or agonistic effect [93]. The oldest form of nutraceuticals known from antiquity, herbals, are the products that comprises either a fresh plant (whole or part), such as dried leaf, roots, fruit, or concentrated extract [97]. Recently, the use of herbals as nutraceuticals has an outstanding impact to promote health benefits and in disease management and prevention. Garlic and ginger are the most commonly used food ingredients that have chemotherapeutic and anti-inflammatory properties. Antioxidant-rich foods, such as ginger, green tea, curcumin, etc., possess effects in weight loss. Moreover, they are also known for their efficacy in NDs [98,99,100].

Fatty acids possess vital beneficial properties in any living organisms. The classification of fat is mainly based on the degree of saturation: saturated fatty acids, monounsaturated fatty acids, and PUFAs. The most significant and common PUFAs are omega-3 and omega-6 PUFAs, both being heavily used as nutraceuticals because of their favorable effects [101,102]. Both probiotics and prebiotics are categorized as nutraceuticals. They are beneficial live microorganisms that promote health, prevent various diseases, and are advantageous for the gastric and intestinal physiology. The consumption of prebiotics and probiotics has been remarkable in the management of gastro-intestinal disorders. Therefore, modern day prebiotics and probiotics are known to be effective in all health problems including NDs and are heavily studied for their therapeutic properties [103,104]. Enzymes or biocatalysts are main functional and structural proteins in the body synthesized by cells. They are responsible for the regulation of body functions and alleviation of health problems. They are economical as they are plant- or animal-based sources and offer numerous advantages upon the consumption of food-based nutraceuticals. Nutraceutical enzymes provide the least benefits in neurological health, but recent therapies have been developed in the management of rare disorders, such as Gaucher disease [105,106].

Non-traditional nutraceuticals include foods obtained from the breeding of agricultural nutrients and products, such as orange juice fortified with minerals, calcium and vitamins in cereals, etc. [42,43,107,108]. Fortified nutraceuticals are also known as designer foods that are enriched from breeding at the agricultural level by nutrients, such as increasing folic acid, calcium, and iron in flour; minerals in cereals; and making milk fortified with cholecalciferol for vitamin D deficiency treatment [109,110,111]. Recombinant nutraceuticals have been formulated from the novel application of biotechnology (genetic engineering or fermentation) in food products [112]. These are the most commonly used categories which are therapeutically advantageous at optimum levels [113], but their production lacks regulations by the Food and Drug Administration [114].

## 5. Nutraceuticals in NDs: Multi-Targeted Avenue

Nutraceuticals in NDs have been able to position themselves as a better and safer integrative approach because naturally occurring compounds have fewer side effects. Moreover, they can be combined with common drug therapy and existing clinical management of NDs to improve the patient’s quality of life in most cases. The effectiveness of several nutraceuticals on multiple pathogenic pathways (involved in NDs) focuses on the rationale behind their use as a complementary therapy in NDs [4,6,115].

**Figure 2:**
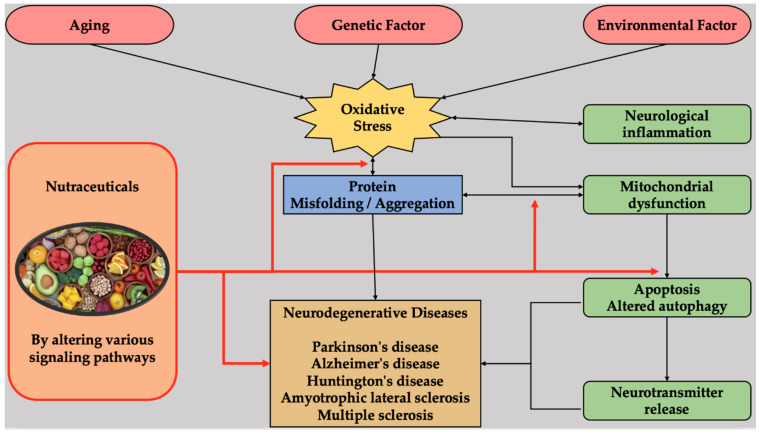
A diagrammatic representation of the cascade involved in NDs and nutraceuticals-mediated multi-targeted approach.

Nutraceuticals work by many mechanisms classified into the following categories: (1) ROS/free radical scavenging and antioxidants; (2) mitochondrial homoeostasis or mitochondria-targeting antioxidants; (3) anti-inflammatory; (4) anti-excitotoxic; (5) anti-apoptotic and caspase inhibitor; (6) modulation of cell signaling pathways; and (7) metal chelation. Instead of adhering to a single pathway or mechanism, most nutraceuticals act through multifaceted mechanistic pathways. Some nutraceuticals, such as coenzyme Q10, resveratrol, astaxanthin, α-lipoic acid, curcumin, and isothiocyanate, to name a few, have shown therapeutic effects against numerous NDs [1,2,4,5,6]. Various studies have also suggested advantageous effects of nutraceuticals against various NDs [116]. Figure 2 presents a diagrammatic representation of the cascade involved in NDs and nutraceuticals-mediated multi-targeted approach.

### 5.1. Nutraceuticals Targeting OS

Myriad nutraceuticals are known to have both direct and indirect impacts by releasing endogenous antioxidants or free radicals/ROS. The induction of cytotoxic proteins in the nuclear factor erythroid 2 (Nrf2)-dependent manners also takes place. The promising therapeutic target identified for NDs is the Nrf2-antioxidant response element (ARE) signaling pathway [117]. In Parkinson’s disease, pre-treatment with isothiocyanate compounds, such as L-sulforaphane, evidently inhibits neuronal death induced by dopamine quinone via decline of ROS accumulation, attenuation of membrane damage, and prevention of DNA fragmentation in mesencephalic dopaminergic neurons and cell lines. Moreover, tert-butylhydroquinone and L-sulforaphane protected astrocytes and neurons against hydrogen peroxide-induced OS in a neuron-astrocyte culture via stimulation of Nrf2-ARE transcriptional pathway [118,119]. Blueberry is a well-recognized nutraceutical in providing neuroprotection through modulation of ROS signaling via MAP-kinase and CREB signaling pathways [120,121]. The neuroprotective impact of resveratrol is also related to its antioxidant properties as well as its ability to upregulate sirtuin1, a longevity-linked gene, and modulate processing of Aβ [122]. Rosmarinic acid and carnosic acid elicited neuroprotective response both in vitro and in vivo by ROS scavenging action [123]. Aged extract of garlic is well-known to protect PC12 cells by repressing ROS generation and reducing caspase-3 activation and DNA fragmentation against Aβ peptide-induced apoptosis [124]. Clove-produced nutraceutical, eugenol, prevented 6-OHDA-induced decline in the dopamine by decreasing lipid peroxidation in the mouse striatum [125]. According to various epidemiological studies, consumption of antioxidant-rich food (such as vitamin E and C) are related with a lower risk of developing NDs including both Parkinson’s disease and Alzheimer’s disease [126].

Furthermore, honey has been shown to reduce brain OS and enhance morphological hippocampus dysfunction in stressed rats, attenuating the cognitive impairment. It is also shown to ameliorate neurodegeneration and OS caused by chronic cerebral hypoperfusion in the midbrain of rat against frequent paraquat exposure [127,128,129]. In a Parkinson’s animal model, a study showed that pretreatment with *Withania somnifera* (ethanolic crude) declined the OS in 6-OHDA-induced rats. In a 3-nitropropionic acid-induced Huntington’s disease mouse model, administration of *Withania somnifera* (root extracts) restored levels of the antioxidant enzymes and attenuated lipid peroxidation [130]. In a rat Alzheimer’s disease model, *Panax ginseng* extract has been shown to enhance the ability of memory and learning, decrease oxidative damage, and prevent the NF-κB and receptors for advanced glycation end product (RAGE) expressions in the hippocampus and cortex of rats induced by advanced glycation end product (AGE) [131].

### 5.2. Nutraceuticals Targeting Mitochondrial Dysfunction

Neurons are susceptible to mitochondrial damage and dysfunction. Various nutraceuticals have been recognized and investigated for their ability to preserve the functions of mitochondria and thus elicit protection against NDs. The turmeric-derived compound curcumin diminished the neurotoxicity induced by 6-OHDA in MES23.5 cells by partly restoring the potential of mitochondrial membrane, enhancing the Cu-Zn SOD action, and repressing increased intracellular ROS and NF-κB translocation [132]. α-lipoic acid (potent stabilizer of mitochondria) shielded neurons in vivo and in vitro against hypoxia, chemotherapy, Aβ, and other neurotoxicity induced by toxicants by maintaining the mitochondrial physiology [133]. Astaxanthin boosted the production of energy and shielded mitochondria of the cultured nerve cells without augmenting ROS generation [134,135]. Another powerful antioxidant for mitochondria is Coenzyme Q10 that shields the neuronal cells by maintaining the mitochondrial functions in striatal excitotoxic lesions caused by the mitochondrial toxin as well as other NDs. Various clinical trials with Coenzyme Q10 recommended its advantageous effects in NDs [136,137]. Such evidence strongly supports the ability of nutraceuticals to fight against the progression and onset of NDs by principally maintaining the mitochondrial homeostasis. Moreover, *Withania somnifera* extract also decreased the increased activity of acetylcholinesterase and restored the activities of mitochondrial ETC complex. In the cortex and striatum of 3-nitropropionic acid-induced rats, attenuated mitochondrial enzyme complex activities and ATP synthesis have been shown to occur by treatment with *Withania somnifera* extract [129].

### 5.3. Nutraceuticals Targeting Neuroinflammation

The anthocyanins are known to act through inhibition of phospholipase A2 that is adversely engaged in a complicated signaling network linking pro-inflammatory cytokines and pro-oxidants to the release of eicosanoid synthesis and arachidonic acid [138,139]. Blueberry exhibited neuroprotection via alteration of inflammation-linked genes expression [120]. Curcumin inhibited NF-κB activation and Aβ-induced cell demise in the cell line of human neuroblastoma, indicating its probable role in the treatment of Alzheimer’s disease [140]. The mustard oil glycoside, curcumin, and green tea flavonoid, epigallocatechin3-gallate, are known to prevent the signaling of pro-inflammatory molecules via toll-like receptors or NF-κB and stabilize BBB in multiple sclerosis [141]. The metabolite of vitamin A, retinoic acid, decreases inflammation and increases tolerance, by maintaining the population of T-lymphocyte in blood, thus it could enhance regeneration, plasticity, behavior, and cognition in multiple sclerosis patients. Vitamin D has also been shown to lessen different inflammatory markers in multiple sclerosis, Alzheimer’s disease, and Parkinson’s disease patients. Moreover, supplementation with vitamin E reduced neuroinflammation and neural degeneration in the rat brain by declining the levels of microglial antigens and astrocytic as well as pro-inflammatory cytokines such as IL-1β and TNF-α [142,143]. Supplementation with omega-3 PUFA reduced the white matter injury and enhanced the neurologic recovery after experimental traumatic injury of the brain by producing protection against loss of myelination, hippocampal neuronal loss, behavioral dysfunction, inflammation, and impulse conductivity [144]. Furthermore, extract of Korean red ginseng also prevented the activation of microglial cells and proinflammatory mediators and increased the activation of p38, NF-κb, JNK, and ERK MAPKs signaling pathways in striatum of a Huntington’s disease-like mice model induced by 3-nitropropionic acid [145]. In animal models of Alzheimer’s disease, studies revealed that vitamin C could reduce OS markers and Aβ formation, and restore behavioral deficits and memory impairment [146].

### 5.4. Nutraceuticals Targeting Apoptosis

Honey is a beehive product which elicits its neuroprotective action against the unfavorable effect of kainic acid via its antioxidant and anti-apoptotic properties, thus preventing neuronal loss and neurodegeneration in the brain. Pretreatment with honey attenuates neuroinflammation, OS, and apoptosis in the cerebellum, cerebral cortex, and brainstem in kainic acid-induced rats [147]. Another bee product, propolis, has been shown to attenuate caspase-3 activity and NO level and inhibit the neuronal loss against excitotoxicity caused by kainic acid in a rat model. These findings indicate that propolis might serve as an antioxidant and anti-apoptotic agent [148,149].

In Parkinson’s animal model (pole test), Korean red ginseng, a perennial herb, could enhance the behavioral impairment of mice and prevent dopaminergic neuronal demise and apoptosis in an MPTP-induced model [150]. In a cellular model, treatment with Korean red ginseng inhibited apoptosis induced by 1-methyl-4-phenylpyridinium ion- (MPP+) on PC12 cells of rat by impeding the cell viability decline and apoptosis via decrease of mRNA expressions of caspase-3 and -9 [151]. Shim et al. showed that *Uncaria rhynchophylla* reduced ROS production and neuronal cell demise as well as prevented the caspase-3 activity in 6-OHDA-induced toxicity (PC12 cells) [152]. A study on seaweed extracts reported increased cell viability, decreased OS, and reduced caspase-3 activity. These findings indicate seaweeds are antioxidant, anti-apoptotic, and neuroprotective agents [153].

Heo et al. reported a compound, diphlor-etho-hydroxy-carmalol, derived from *Ishige foliacea*, which increased the cell viability and inhibited caspase-9 and caspase-3, imparting anti-apoptotic activity [154]. Crocin is the carotenoid compound obtained from *Crocus sativus*, which is known to exhibit neuroprotective effects in different CNS disorders in vivo and in vitro as well as decrease the expressions of binding-immunoglobulin protein (BiP) and CHOP, thereby inhibiting the pro-apoptotic factor caspase-12 activation in PC12 cells [155,156]. The natural phytoestrogen resveratrol improves the autophagic degradation of α-Syn in PC12 cells via induction of AMP activated protein kinase of the mammalian silent information regulator 1 signaling pathway [157].

The apoptotic pathways and induction of autophagy signifies an essential avenue in the therapeutic α-Syn targeting, so resveratrol is a potential compound for this approach, even though numerous factors, such as its solubility and stability at gastric pH, increased metabolic turnover, and gut microflora can compromise the bioavailability of polyphenolic compounds in the brain [158].

Nutraceuticals and their relevance in NDs are summarized in Table 1.

### 5.5. Nutraceuticals Targeting Calcium Overload and Excitotoxicity

Blueberry extract has been shown to impart neuroprotection against Aβ, which is mediated by antagonizing aggregated Aβ-induced ATP leakage and increased intracellular Ca^2+^ [179]. Despite being an effective therapeutic strategy, few studies are available that assess the ability of nutraceuticals to prevent and antagonize intracellular overload of Ca^2+^ [180]. The extract of *Uncaria rhynchophylla* elicits the effect of free radical scavenging and inhibits the lipid peroxidation in a kainic acid-induced excitotoxicity rat model [181].

Isolated from ethyl acetate portion of the *Eisenia bicyclis* (brown algae) methanol extract, phloro-fucofuroeckol A, phloro-tannins eckol, and 7-phloroeckol exhibit their neuroprotective effect via reduction of intracellular ROS production and intracellular Ca^2+^ level in Aβ-induced cytotoxicity in PC12 cells. Diphlor etho-hydroxy-carmalol is also reported to attenuate ROS generation as well as inhibit intracellular levels of Ca^2+^ [182].

## 6. Conclusions and Futuristic Approaches

NDs are a group of debilitating disorders that progressively rob a person of their essential bodily functions. Although extreme efforts have been put for the advancement of therapeutic strategies for these NDs, the conventional treatment interventions are unfortunately accompanied by various undesirable effects over a period of time despite their ability to offer symptomatic relief.

Luckily, with the advent of nutraceuticals, a complementary therapeutic avenue in tackling of these apparently evasive problem of NDs has been offered. By virtue of their origin from natural sources, nutraceuticals seem to be a favorable treatment and complementary approach with the existing therapeutic options, as harnessing the naturally available treatment strategies might avoid undesirable effects. Recently, extensive studies endeavored to elucidate the growing interest of nutraceuticals as a collective approach to the standard pharmacological therapy, affecting one or more of the aforementioned pathogenic processes or pathways.

In fact, several nutraceuticals mentioned in this manuscript have been presented to not only be preventive and complementary but also therapeutic for NDs. Significantly, we highlight in this review how nutraceuticals possibly target the different pathogenic processes such as neuroinflammation, OS, mitochondrial dysfunction, protein misfolding, excitotoxicity, and apoptosis to rescue pathological outcomes in NDs such as Huntington’s disease, amyotrophic lateral sclerosis, multiple sclerosis, Alzheimer’s disease, and Parkinson’s disease. Moreover, the neuroprotective potentials of nutraceuticals against NDs were supported by extensive experimental studies. In fact, it is becoming evident that multi-target approaches, rather than single-drug options should be considered, varying the ND etiology and progression.

Notwithstanding this, the acknowledgement that nutraceuticals could be of therapeutic value bids innumerable opportunities and new scenarios to study and explore other natural compounds which have not been considered in terms of their potential actions of neuroprotection in NDs. To make the best use and maximize the neuroprotective effects against NDs, the delivery of nutraceuticals could also be optimized. As an adieu note, it is encouraged to imagine that we might be able to transform the diet in order to mitigate or prevent the NDs’ progression.

## Figures and Tables

**Table 1:** Summary of nutraceuticals and their relevance in NDs.

Nutraceuticals	Targeted Mechanism(s) and Pathway(s)	Elicited Effect(s)	References
Coenzyme Q10	Preserved functions of mitochondria	Protection against various excitotoxic lesions of striatum generated by the mitochondrial inhibitor (Complex II mitochondrial toxin) and various NDs.	[135,136]
Vitamin D3	-	Stabilizes and alleviates symptoms of PD without stimulating hypercalcemia	[159]
L-sulforaphane (isothiocyanate compound)	Inhibition of pro-inflammatory signaling via toll-like receptors or NF-κb and decrease in ROS.	Inhibited neuronal death induced by dopamine quinone and stabilized the BBB in MS.	[118,119]
Vitamin D	Anti-inflammatory mechanism	Protective in AD, MS, and PD patients.	[143]
Vitamin B6		Amelioration of PD symptoms	[160,161]
Epigallocatechin-3-gallate, mustard oil (glycoside)	Prevention of pro-inflammatory signaling via NF-κB or toll-like receptors	Stabilized the BBB in MS	[141]
Creatine	-	Ameliorates PD symptoms	[162]
CoQ10, tocopherol, and GSH	-	Shows a small yet significant betterment in PD symptoms	[163,164]
Curcumin	Restoration of mitochondrial membrane potential, suppression of increased intracellular ROS, inhibition of pro-inflammatory signaling via toll-like receptors and NF-κb, decreased malondialdehyde, cytochrome c levels, and cleaved caspase-3 expression	Ameliorated neurotoxicity induced by 6-OHDA in MES23.5 cells, stabilized the BBB in MS, protected SH-SY5Y cells against MPTP-induced dopaminergic neurotoxicity or MPP (+)	[132,165,166,167]
Astaxanthin	Enhancing production of energy by shielding mitochondria	Protection of cultured nerve cells	[134,135]
Carnosic acid and Rosmarinic acid	Scavenging of ROS	Neuroprotective effect in both in vivo models of neurodegeneration and in vitro neuronal death models and protected neuronal cells from oxidative stress induced by hydrogen peroxide.	[123,168]
Eugenol	Elevated GSH level and reduced 6-OHDA-induced lipid peroxidation	Prevented 6-OHDA induced decrease in the dopamine level in the striatum of mouse	[125]
Tert-butyl hydroquinone	Stimulation of Nrf2-ARE transcriptional pathway	Protected astrocytes and neurons against hydrogen peroxide- induced OS	[118]
Aged garlic extract	Inhibiting generation of ROS and reduced activation of caspase-3, DNA fragmentation and cleavage of PARP.	Shielded PC12 cells against apoptosis induced by Aβ peptide	[124]
Anthocyanins	Negative regulation of pro-oxidants and -inflammatory cytokine signaling pathways	Neuroprotection	[121,139]
Apigenin	Stabilized the potential of mitochondrial membrane	Modulated glutamatergic and GABAergic transmission and protected neurons against Aβ-mediated toxicity induced by copper	[169,170]
Isothiocyanates (e.g., sulforaphane)	Activate Nrf2/ARE pathway	Promoted the upregulation of GSH, upregulation of antioxidant enzymes through activation of Nrf2, prevention of apoptosis mediated by Aβ	[171,172]
Soy isoflavones	Modulation of brain cholinergic system	Amelioration of age-associated cognition decline and neuronal loss in male rats	[173]
Vitamin E	Reduction of pro-inflammatory cytokines such as TNF-α and IL-1β	Reduction in neuronal degeneration and neuroinflammation in the rat brain	[174]
Folate, vitamin B6 and B12	Downregulation of GSK-3β, JNK, ERK, and p38MAPK pathway	Inhibit memory deficits and tau hyperphosphorylation induced by homocysteine administration and decrease memory deficits.	[175,176]
Extracts of tangerine peel (rich in polymethoxylated flavones), red clover (rich in isoflavones), and cocoa-2 (rich in procyanidins)	-	Decreases dopaminergic neuronal loss	[177]
Ginkgo biloba extract 761 (EGb 761, comprising 6% terpenoids 24% flavonoids)	-	Antioxidant effect, promotes neurorecovery of damaged dopaminergic neurons of midbrain, enhances locomotion	[178]

6-OHDA, 6-hydroxydopamine; AD, Alzheimer’s disease; ARE, antioxidant response element; BBB, blood brain barrier; DNA, deoxyribonucleic acid, ERK, extracellular-signal-regulated kinase; GSH, glutathione; GSK-3β, glycogen synthase kinase 3; IL-1β, interleukin-1β; JNK, c-Jun N-terminal kinase; MPTP, 1-methyl-4-phenyl-1,2,3,6-tetrahydropyridineMS; Multiple sclerosis; ND, neurodegenerative disease; NF-κb, nuclear factor kappa light chain enhancer of activated B cells; Nrf2, nuclear factor erythroid 2-related factor 2; p38MAPK, p38 mitogen-activated protein kinase; PD, Parkinson’s disease; ROS, reactive oxygen species; PARP, poly-ADP ribose polymerase; TNF-α, tumor necrosis factor, MES23.5 cells, a dopaminergic cell line; SY5Y cells, human derived cell line.
